# Age, brood fate, and territory quality affect nest-site fidelity in white stork *Ciconia ciconia*

**DOI:** 10.1186/s12983-023-00506-y

**Published:** 2023-09-21

**Authors:** Joanna T. Bialas, Joachim Siekiera, Artur Siekiera, Wiesław Chromik, Łukasz Dylewski, Marcin Tobolka

**Affiliations:** 1 Department of Zoology, Poznań University of Life Sciences, Wojska Polskiego 71C, 60-625 Poznan, Poland; 2 Żywocice, Poland; 3 Katowice, Poland; 4 Konrad Lorenz Institute of Ethology, University of Veterinary Medicine Vienna, Savoyenstraβe 1a, 1160 Vienna, Austria

**Keywords:** Site fidelity, Nest fidelity, Nest switching, Breeding dispersal, Ring resighting

## Abstract

**Background:**

A particular type of site fidelity is faithfulness to the nest site, where birds are not only reoccupying breeding territories but also reusing nests built in previous breeding seasons. Staying faithful to the nest site is believed to be an adaptive strategy, and based on the ability to predict an individual's own breeding success, a hypothesis of “win-stay:loose-switch” was proposed. In this study, we aimed to resolve which factors affect the nest-site fidelity of white stork *Ciconia ciconia*, species known for reusing nests available in the breeding sites. Basing on ring recoveries from 31 years of studies in Western and Southern Poland, we analysed the impact of intrinsic and extrinsic factors on nest-site fidelity.

**Results:**

We found that increasing age and breeding success (i.e. producing any fledglings or not) increased the probability of reusing the nest, but in the oldest individuals, the probability decreased. In turn, the probability of breeding success increased with age, the increasing number of reproductive events on the particular nest, and the presence on the nest in the previous year. However, the oldest individuals had lower probability of success, as the relationship was curvilinear. The number of fledglings, however, was influenced only by an individual's age. The number of reproductive events on the nest was, in turn, affected by age, with the youngest and oldest individuals using the current nest for the least number of years.

**Conclusions:**

Our study shows that the decision process of whether to stay faithful to the nest or switch is based on the experience from the previous breeding event, consistently with the “win-stay:loose-switch” hypothesis. Our results also show that site fidelity benefits white storks, as the probability of breeding success increases if the nest is reused. Results also show the senescence effect that lowers breeding success and site fidelity probabilities.

**Supplementary Information:**

The online version contains supplementary material available at 10.1186/s12983-023-00506-y.

## Introduction

One of the main benefits of staying faithful to the breeding site is habitat familiarity [1–4]. It gives an advantage over competitors [5], makes exploration of the habitat more efficient [6], and therefore enables optimal use of food resources [7–9] and also facilitates predator avoidance [10, 11] even in suboptimal habitats. Whilst predator avoidance was suggested as the main factor in habitat selection [12], it is worth mentioning that not all species are particularly threatened by predation, e.g. the white stork *Ciconia ciconia* [13].

One of the earliest examples of site fidelity is a study on the Great Tits *Parus major,* which remained faithful to suboptimal habitats even though optimal habitats were available [14]. Up to date, there are plenty of studies showing cases of species both remaining faithful regardless of territory quality [1, 15–18] or switching sites [19–23]. Although the quality of the territory seems to be crucial in habitat selection, there are other factors involved in the decision process of whether to remain faithful or switch sites. We can divide these factors into environmental and individual [24]. Environmental characteristics affecting site fidelity are mainly habitat stability [1, 25], predictability of reproductive failure [26], variability in territory quality within a habitat [1, 21, 27, 28], and population pressure [20]. Individual factors, on the other hand, would be a previous reproductive success [29, 30], age [29], or habitat familiarity [2, 3].

Other functions proposed to explain site fidelity are strengthening pair bonds and, therefore, mate retention if remaining faithful [31, 32] or reducing rates of ectoparasitism if switching [33, 34].

Although it is advantageous to remain faithful, and the cost of switching sites can be high, it may also be favourable to switch. Individuals decide whether to remain site faithful according to one of three rules of thumb: (1) win‐stay:lose‐switch (WSLS) rule [24, 27, 35]—they return to the sites of the successful reproduction, or they do not if they failed [3], (2) always-stay rule—they stay faithful regardless of the breeding output [14, 24] or (3) always-switch where site fidelity is a suboptimal form of habitat selection [24]. Individuals may use the first decision process (WSLS) when habitat varies spatially, but not temporarily in quality, and assessing potential fitness is impossible for an individual in any other manner than to use its knowledge about past breeding experiences [24, 36]. However, if the habitat is temporarily unpredictable, decisions would not be affected by the breeding experience. Individuals would choose to always stay when the habitat is homogenous or always switch between the habitat patches when it is heterogonous [1, 35]. The predictability of the breeding effect may differ depending on the factors influencing it. One such factor is nest predation which may be predictable yearly if the predator is always present in the area [37, 38]. On the other hand, environmental changes such as floods can make habitat quality unpredictable [24].

Many of the studies of site fidelity, particularly in birds, focus on individual factors. For instance, different strategies for males and females may arise from differences in parental investment [32, 39]. Many studies of birds showed female-biased breeding and natal dispersal [39, 40] and higher fidelity to the site of males than females [41], as males are usually responsible for establishing and defending territories. Another individual factor that may influence the decision process is age, i.e. site fidelity may increase with age and breeding experience as older birds tend to be more socially dominant and of higher quality [41, 42]. Site fidelity may increase with the number of breeding attempts [43] due to increasing site familiarity; therefore, switching sites may be disadvantageous. However, such factors as age, years of prior residence, or breeding output are challenging to disentangle from each other because they are often correlated. Environmental factors that were shown to be connected with site fidelity include breeding density, habitat or nest characteristics [24, 42, 44–47].

In this study, we focus on the white stork as an example of a particularly interesting species to study site fidelity. Storks are known to reuse nests yearly, and nest-site fidelity has been reported in over 80% of individuals [29, 48]. Switching nests was observed to be age-related, more frequent in young individuals, and usually followed by mate changes [49]. Vergara et al. [29] have shown that the main factors influencing whether an individual remains faithful to its nest are the previous year's breeding effect and the age of a breeder. However, these results have been obtained from the Western European population of the white stork, which is very specific in terms of its ecology. It has become more independent from the original wintering grounds due to the behaviour of foraging at open landfills [50, 51], which affected the migratory behaviour [52, 53]. Instead, the central-eastern population of the species remains fully migratory, performing the longest yearly migration within all subpopulations of the white stork [54]. There is also a difference in breeding ecology, i.e. white storks from the western population are often colonial breeders, while white storks are solitary nesters in the studied central-eastern population. Studies from the Central-Eastern European population of white stork show that site fidelity is related to age but is also year-dependent [55]. The authors suggested a connection between population density and site fidelity [55]. However, the colony size was not connected to site fidelity in the western population [29].

Therefore, having an opportunity to study a solitary nesting population of the white stork, with separated territories differing in land cover and microhabitats, contrasting to previous studies on this species, this research aimed to study which factors influence nest-site fidelity. We tested whether white storks use the simple decision-making process “win-stay:loose-switch” or whether nest-site fidelity depends on any other factors like age or sex but also the quality of the territory and weather conditions. We hypothesize, based on previous studies, that (1) age and previous breeding success are the main factors influencing nest-site fidelity, (2) territory quality influences site-fidelity, (3) site fidelity is affected by the general breeding effect of the population, assuming that storks may be able to recognize neighbours’ breeding output, (4) reproductive success depends on years spent on the same nest as familiarity with the territory increases, (5) number of fledglings produced depend on age of an individual, 6) number of reproductive events are correlated with age of an individual.

## Results

**Fig. 1:**
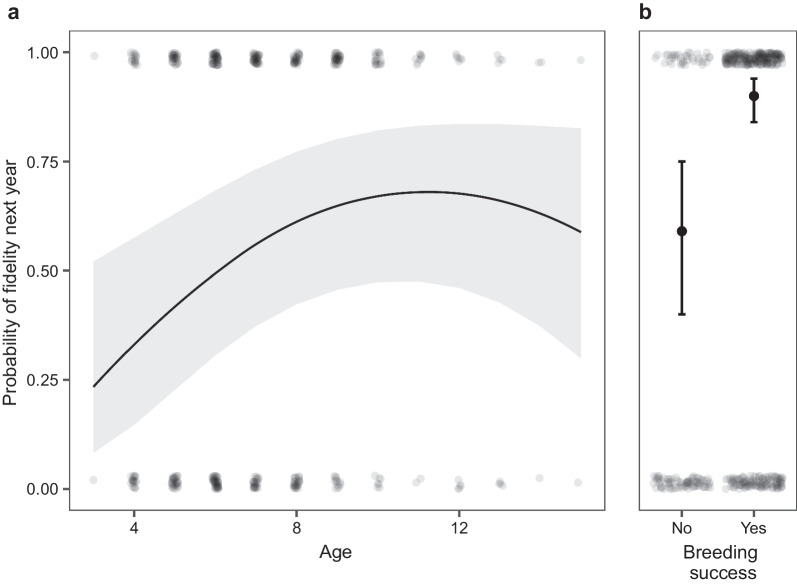
Effects of individual’s age (**a**), and breeding success **b** on the probability of nest-site fidelity in the next year. Predictions resulted from generalized mixed model with binomial distribution. Transparent dots are observed values, in **a** line indicates predictions from the model and grey areas represent 95% CI, in **b** solid dots indicate predictions from the model and whiskers represent 95% CI. Predictions in **a** are calculated for limited range of age classes (3–15)

We observed nest-site fidelity in 546 cases (78.11%) of 699 records (Table 1). Model GLMM_1 (N = 565) shows that the probability of being faithful to the nest is influenced by an individual's age and breeding success in the previous year (Fig. 1, Additional file 1: Fig. S1, Table 2). The relation of the site-fidelity probability and age was quadratic, with young and old individuals being the least faithful (linear term: 0.64 ± 0.23, p = 0.01; quadratic term: − 0.03 ± 0.01, p = 0.01). Successful individuals were more likely to reuse the nest in the next year (1.85 ± 0.48, p < 0.01). We included results of the same model from which we excluded sex of an individual in the (Additional file 1: Table S1). In results of this model two additional variables have significant positive influence on site fidelity: pastures (3.80 ± 1.73, p = 0.03) and other agricultural lands (4.10 ± 2.08, p = 0.05).

**Table 1:** Summary of the overall number of observations of fidelity or switching nests by individually marked breeding white storks

Sex	No. observed switching	No. observed fidelity	Total
Females	62 (45)	248 (79)	310 (110)
Males	64 (46)	241 (86)	305 (113)
Undetermined	27 (23)	57 (45)	84 (108)
Total	153 (114)	546 (210)	699 (331)

The numbers outside the brackets indicate all observations regardless of bird ID, and the numbers inside the brackets indicate different individuals

**Table 2:** Results of GLMM for probability of fidelity to the nest in the next year

	Estimate	SE	p
(Intercept)	− 2.10	3.82	0.58
Age	0.64	0.23	**0.01**
Age^2	− 0.03	0.01	**0.01**
Breeding_success	1.85	0.48	**0.00**
Sex	− 0.01	0.25	0.96
No_repr_events	0.17	0.14	0.24
Relative_productivity	0.02	0.17	0.89
Hydro_network	− 0.17	0.28	0.55
Human_altered	3.67	2.05	0.07
Arable_land	1.04	0.95	0.27
Pastures	2.80	1.96	0.15
Other_agri_lands	3.54	2.29	0.12
PPT_av	0.00	0.01	0.71
TMIN_av	− 0.09	0.20	0.66

The explanatory variables were age and its quadratic term (Age, Age^2 respectively), breeding success (Breeding_success), sex of an individual, number of reproductive events of an individual on the particular nest (No_repr_events), productivity relative to the population mean (Relative_productivity), natural logarithm of length of hydrological network (Hydro_network), cover of human-altered habitat (Human_altered), cover of arable lands (Arable_land), cover of pastures and meadows (Pastures), cover of other agricultural lands (Other_agri_lands), average precipitation (PPT_av) and minimum temperature (TMIN_av) during the breeding season. For binary variables breeding success and sex the presented values are for consecutively presence of success and males, as lack of success and females are treated as a reference (the estimates are equal to 0). Significant results (p < 0.05) are in bold

**Fig. 2:**
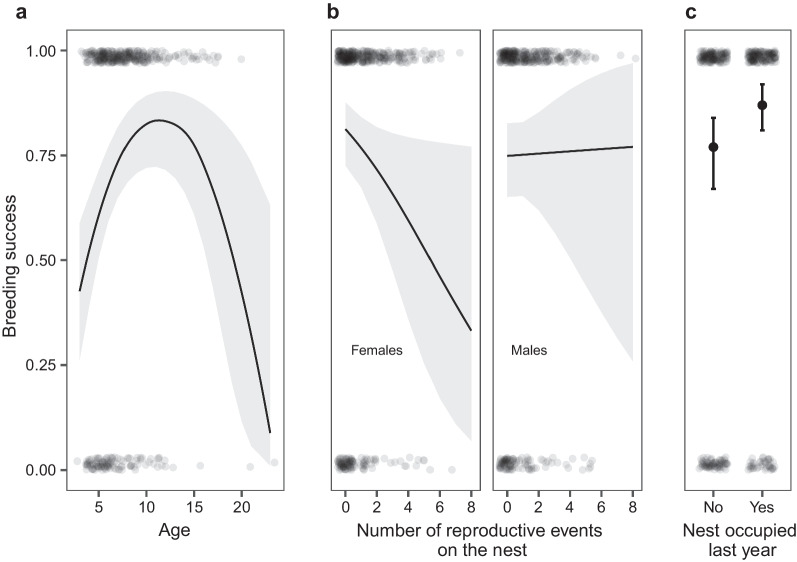
Effects of individual’s age (**a**), number of reproductive events on the nest in interaction with individual’s sex (**b**), and individual’s presence on the nest in the previous year (**c**) on the probability of breeding success. Predictions resulted from generalized mixed model with binomial distribution. Transparent dots are observed values, in **a** and **b** lines indicate predictions from the model and grey areas represent 95% CI, in **c** solid dots indicate predictions from the model and whiskers represent 95% CI

Model GLMM_2 (N = 727) shows that the breeding success is influenced by age, the number of reproductive events on the particular nest, and the presence on the nest in the previous year (Fig. 2, Table 3). The number of reproductive events reduce the breeding success for females, whereas there is no effect for males (0.29 ± 0.14, p = 0.04). Similar to nest-site fidelity, the relationship between breeding success and age is quadratic, with the youngest and the oldest individuals having the lowest probability of breeding success (linear term: 0.62 ± 0.16, p < 0.01; quadratic term: − 0.03 ± 0.01, p < 0.01). The probability of breeding success also increases when the bird has been breeding on the same nest the year before (0.71 ± 0.31, p = 0.02).

**Table 3:** Results of GLMM for breeding success and LMM for number of fledglings

	Breeding success			Number of fledglings		
	Estimate	SE	p	Estimate	SE	p
(Intercept)	− 0.74	3.32	0.82	− 0.17	1.55	0.91
Nest_last_year	0.72	0.31	**0.02**	− 0.04	0.12	0.72
Age	0.62	0.16	**0.00**	0.34	0.08	**0.00**
Age^2	− 0.03	0.01	**0.00**	− 0.01	0.00	**0.00**
Sex	− 0.38	0.25	0.13	0.05	0.11	0.63
No_repr_events	− 0.27	0.13	**0.04**	0.00	0.05	0.98
Hydro_network	− 0.19	0.25	0.45	0.01	0.10	0.91
Human_altered	0.13	1.61	0.93	− 0.28	0.65	0.66
Arable_land	− 0.80	0.84	0.34	0.45	0.34	0.19
Pastures	2.35	1.73	0.17	− 0.19	0.60	0.76
Other_agri_lands	0.12	1.92	0.95	0.33	0.87	0.70
PPT_av	− 0.01	0.01	0.16	0.00	0.00	0.21
TMIN_av	0.24	0.17	0.14	0.06	0.10	0.57
Sex:No_repr_events	0.29	0.14	**0.04**	− 0.05	0.05	0.38

The explanatory variables were occupancy of the same nest in the previous year by an individual (Nest_last_year), age and its quadratic term (Age, Age^2 respectively), sex of an individual, number of reproductive events of an individual on the particular nest (No_repr_events), natural logarithm of length of hydrological network (Hydro_network), cover of human-altered habitat (Human_altered), cover of arable lands (Arable_land), cover of pastures and meadows (Pastures), cover of other agricultural lands (Other_agri_lands), average precipitation (PPT_av) and minimum temperature (TMIN_av) during the breeding season and interaction between sex and number of reproductive events (Sex:No_repr_events). For binary variables Nest_last_year and Sex the presented values are for consecutively occupied nests and males, as unoccupied nests and females are treated as a reference (the estimates are equal to 0). Significant results (p < 0.05) are in bold

**Fig. 3:**
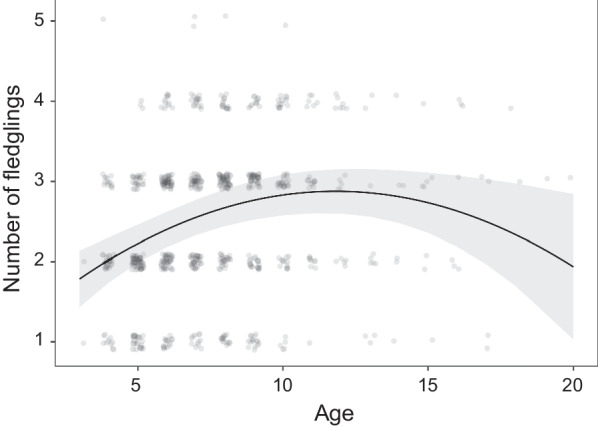
Effect of individual’s age on the number of fledglings it produced. Predictions resulted from linear mixed model. Transparent dots are observed values, line indicates predictions from the model and grey area represents 95% CI

Model LMM_3 (N = 542) shows that the number of fledglings is affected only by age (Fig. 3, Table 3), and as for all three models, the relationship is quadratic, with the youngest and oldest birds producing the lowest number of fledglings (linear term: 0.34 ± 0.08, p < 0.01; quadratic term: -0.01 ± 0.00, p < 0.01).

**Fig. 4:**
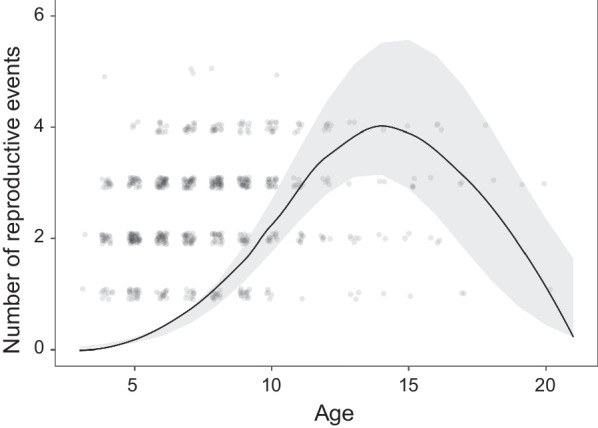
Effect of individual’s age on the number of reproductive events. Predictions resulted from generalized linear mixed model. Transparent dots are observed values, line indicates predictions from the model and grey area represents 95% CI

Model GLMM_4 (N = 727), shows that the number of reproductive events on the nest is also affected only by age (Fig. 4, Table 4), and as for all previous models, the relationship is quadratic, with the youngest and oldest birds staying on the current nest for less years (linear term: 1.12 ± 0.08, p < 0.01; quadratic term: − 0.04 ± 0.00, p < 0.01).

**Table 4:** Results of GLMM for number reproductive events on the nest

	Estimate	SE	p
(Intercept)	− 8.27	1.92	0.00
Age	1.12	0.08	**0.00**
Age^2	− 0.04	0.00	**0.00**
Sex	0.01	0.13	0.96
Hydro_network	0.16	0.16	0.34
Human_altered	1.23	0.90	0.17
Arable_land	0.14	0.52	0.79
Pastures	− 0.45	0.92	0.63
Other_agri_lands	0.30	1.04	0.77

The explanatory variables were age and its quadratic term (Age, Age^2 respectively), breeding success (Breeding_success), sex of an individual, number of reproductive events of an individual on the particular nest (No_repr_events), productivity relative to the population mean (Relative_productivity), natural logarithm of length of hydrological network (Hydro_network), cover of human-altered habitat (Human_altered), cover of arable lands (Arable_land), cover of pastures and meadows (Pastures), cover of other agricultural lands (Other_agri_lands). For binary variable sex the presented value is for presence of males, as females are treated as a reference (the estimate is equal to 0). Significant results (p < 0.05) are in bold

## Discussion

Our results show that the probability of being faithful to the nest is influenced by the age of an individual and breeding success in the previous year. The breeding success itself, however, is influenced by age of breeder, the number of reproductive events of individual on the particular nest, and it’s presence on the nest in the preceding year. The number of fledglings is influenced only by parent's age. The number of reproductive events on the nest is also affected only by age of an individual. These results show that white storks’ decision process whether to remain faithful to the nest or switch is in line with the strategy of win-stay:lose-switch, which is well documented in many bird species [28, 46, 69, 70]. In case of the white stork, where there is hardly any brood parasitism [71], and nest predation is also rare [13], the theory proposed by Switzer [24] seems to match the actual strategy used by storks in the studied population.

An individual's age was also related to whether the bird stayed or switched, which is probably connected to the fact that age also affects breeding success in the same pattern, as more experienced birds were more effective breeders. Although the breeding success of birds has been shown to increase with age and breeding experience [72, 73], the relationship between age and fidelity may instead be a by-product of the employment of win-stay:loose-switch strategy [30] as first breeding attempts of young individuals usually fail [42, 74]. On the other hand, older individuals are less productive due to the effects of ageing [75, 76], and their survival probabilities also decrease with age [77]. It has been shown that although an individual's age and breeding experience are related to prior residence years, the latter directly influences site fidelity [30]. In our study we also show, that the number of reproductive events on the nest depends on age of an individual, however the relationship is not linear, with the youngest and the oldest individuals having spent the least number of years on the current nest. A previous study of the Western European population of white storks [29] also showed that the factors influencing nest-site fidelity are age and breeding success. The breeding success was therefore suggested to be linked to an individual's age due to its experience. It has been shown that younger individuals not only occupy low-quality nest sites but also use resources less efficiently [29, 49], leading to a decrease in breeding success probability. Although both variables—age and breeding success were significantly affecting the probability of fidelity, the pattern of staying faithful while being successful was visible throughout all age classes. However, it was weaker in the youngest (in the 3rd year of life) and oldest birds (in > the 10th year of life), most likely because of the smaller sample size in these age groups. This is, however, opposite to the findings of Vergara et al*.* [29], that found differences between age classes in fidelity regardless of the breeding success. Moreover, we tested the influence of the interaction between age and breeding success on site fidelity. Not only was it not significant (and therefore excluded from the final model), but across all age classes, we observed that successful breeders tend to stay faithful to the nest. Therefore, consistently with Bai & Severinghaus's [30] findings in Ryukyu Scops Owl *Otus elegans botelensis*, we conclude that in the Central-Eastern European population of white stork it is the breeding success that directly influences site fidelity regardless of the age of an individual.

Our results of the site fidelity pattern are consistent with the results of the factors affecting breeding success in the studied population. Breeding success was determined by the age of an individual but also by whether the bird was using the nest in the previous breeding season and the overall number of reproductive events on the nest in interaction with sex. This suggests that in the case of the studied population, the most critical predictor of breeding success, besides age, is habitat familiarity. However, the overall number of years spent on the nest affects the probability of breeding success differently for males and females. In males, an increase in the years spent on a particular nest increases the probability of breeding success, but the opposite is true for females. This may arise simply from the process of ageing as the white stork, like other long-lived species, may reduce reproductive abilities after the age of high fertility [78, 79]. Although both pair members are involved in incubation and chick-rearing processes [49] it is the female that invests in egg laying, which is physiologically demanding. On the other hand, white storks arrive earlier to the breeding grounds along with lifetime experience until they reach a particular age, and males arrive earlier than females. When they are older, the difference in arrival times between males and females blur [80]. That may, in turn, cause laying unfertile eggs when the female arrives earlier than the male and spring turnover during arrivals [81, 82], which we also observed in our study. The number of fledglings, on the other hand, is influenced only by an individual's age.

The influence of senescence is visible throughout all of our result. The oldest fraction of birds have lower reproductive success, therefore they also show lower site fidelity and lower number of reproductive events on the currently occupied nest. This may arise from the lower number of records of the oldest birds (Fig. 2, [83]), however, in long-lived species the effect of senescence, as mentioned, is well-documented [78, 79, 84, 85].

When excluding sex from the models (Additional file 1: Table S1) we also found an effect of habitat on site fidelity—the probability of remaining faithful to the nest increased with an increasing share of pastures and meadows, and other agricultural lands, which are agricultural lands with complex structures rich in natural or semi-natural vegetation. This is consistent with previous findings where we and other authors showed that in these particular habitat, storks tend to have higher breeding success [60, 61, 86]. The previous study of white storks’ nest-site fidelity [29] did not show any relationship between staying faithful to the nest and habitat. However, as the authors stated, most studied colonies had access to landfills for the entire breeding season. Unless waste management is not changing in the following years, we expect a similar pattern to occur, at least in the case of nests that have direct access to landfills in Poland. We have previously shown a growing trend towards nesting closer to landfills among the white stork population in Poland [60, 86]. Prey-switching behaviour was suggested to reduce maladaptive consequences of site fidelity in a rapidly changing environment [87, 88].

Nevertheless, this study did not find any relationship between breeding success, the number of fledglings or the number of reproductive events on the nest and habitat structure. Thus, there may be other independent factors affecting the decision whether to remain faithful to the nest. Interestingly, we did not find any effect of weather conditions on fidelity, breeding success, or productivity. This is inconsistent with the previous studies showing a relationship between breeding success and weather conditions—both minimum temperature and precipitation [58]. This may suggest that the prior residence is highly beneficial for both breeding success and, thus, indirectly for fidelity. The value asymmetry hypothesis could explain this phenomenon [89, 90]. Although habitat familiarity in the case of white stork does not necessarily reduce predation risk, which is low whatsoever, it may greatly influence foraging efficiency and give the residents an advantage over potential intruders and competitors. At the same time, neighbours are known individuals, therefore, the cost of territory defence is also lower [91]. Thus, previous owners may be more engaged and are more likely to win contests against newcomers as they perceive the value of the territory [92–94].

We did not discover any link between sex and nest site fidelity. In previous studies by Vergara et al. [29], sex showed a marginally significant effect, with females more prone to switch between nests than males. Although, the interaction between productivity and sex significantly influenced nest-site fidelity, with more productive males being more prone to stay. The authors suggested that the significant interaction could indicate that the sexes enter the reproductive population at different ages. Younger individuals were shown to arrive later at the breeding grounds [48, 80, 95], and therefore they have to choose among the available nests potentially in suboptimal territories or even establish new nests that are less frequently reoccupied [96]. Moreover, males tend to arrive before females on the breeding grounds [48], and according to Vergara et al. [29], it could explain the weak sex differences in nest-site fidelity. In our study, however, neither sex nor interaction between sex and relative productivity has shown the slightest effect on nest-site fidelity. It may arise from the fact that the costs of reproduction are shared by both parents in white storks, except for the cost of egg production. white stork is a species that cooperates during the whole breeding season—both parents incubate eggs, and after hatching, both feed the offspring [49]. Therefore, because the investment of both sexes is similar, we do not expect differences in decision-making strategies between males and females.

We did not find any relationship between remaining faithful to the nest and relative number of fledglings, i.e. number of fledglings produced by particular pair reduced by the mean number of fledglings in the population. It is consistent with the fact that we did not find relationship between productivity and any measurements of fidelity (if the nest was occupied last year or the number of years spent on the nest before). Taken together these suggest that the number of fledglings produced does not affect fidelity at all, and just a successful breeding (i.e. producing at least one fledgling) is enough for staying faithful to the nest. Similarly in Ryukyu Scops Owl, it was shown that relative productivity did not affect the decision whether to stay or switch the nesting site [30].

Our data were insufficient to study the relationship between nest site and mate fidelity due to the low number of pairs with both partners being banded (40 records during the whole study period). However, as white storks are known to change mates after switching between nests [49], and the reproductive success of mating with a new partner is reduced [97, 98], it adds up to the costs of nets switching. As Vergara et al. [29] showed, the consequences of nest switching are related to the higher probability of breeding failure. The causes of these failures, especially those related to divorces, should be thoroughly studied in the future. Disentangling mate and site selection could shed more light on the factors and underlying mechanisms that influence both.

## Conclusions

Our study shows that the decision process of whether to stay faithful to the nest or switch is based on the experience from the previous breeding event. Breeding success in the previous year and age of an individual, as well as habitat quality, regardless of the sex of an individual, affect the site fidelity. Our results also show that site fidelity benefits white storks, as the probability of breeding success increases if the nest is reused. While facing rapid changes in the environment, the strategy of win-stay:loose-switch that white storks employed suggests greater behavioural flexibility of this species and may result in the capability for adjusting their behaviour as a result of individual or social information [88, 99].

## Methods

### Data collection

**Fig. 5:**
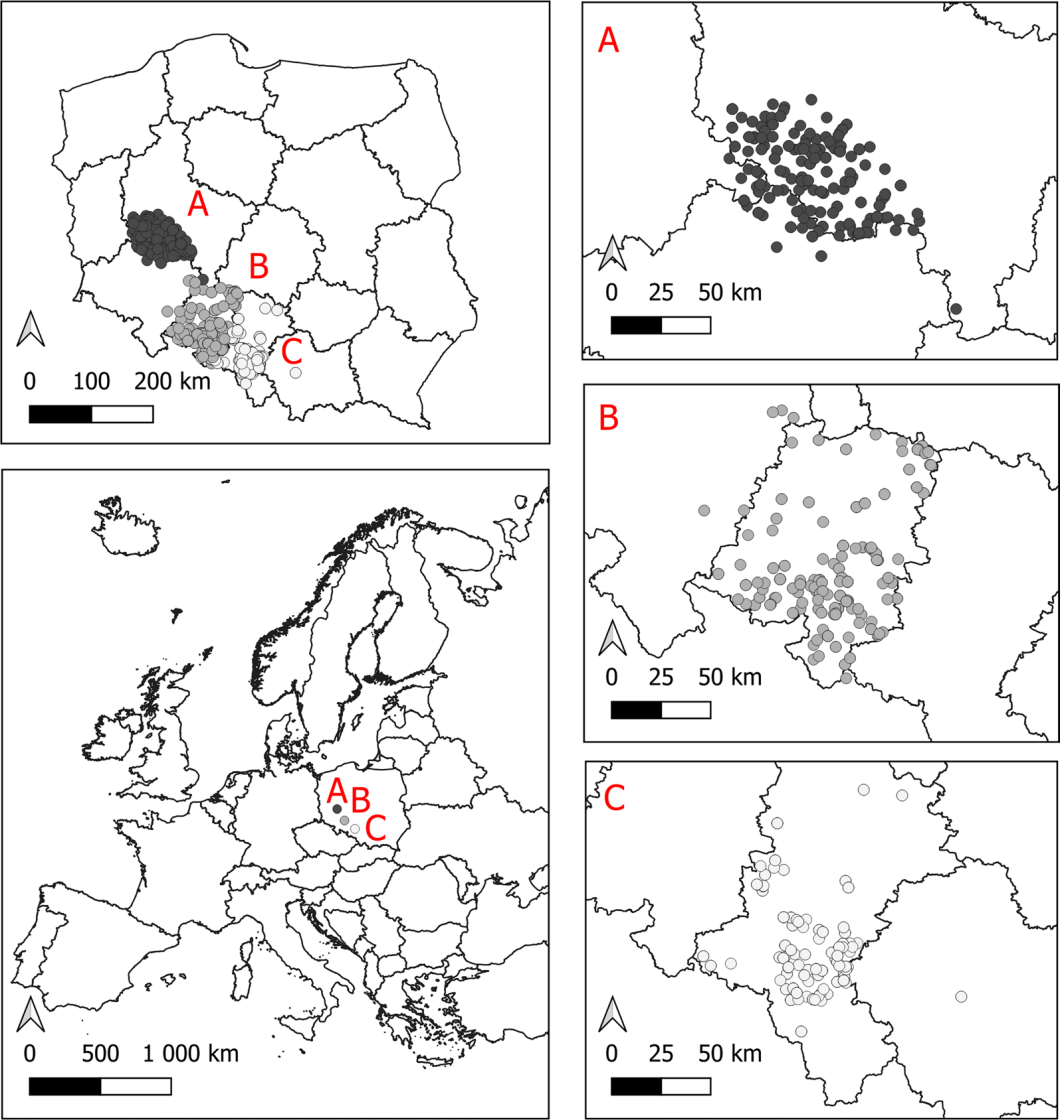
Map of white storks’ nests on three study areas. The lower left map shows the location of study areas in Europe, and upper left map shows the location in Poland. Circles in dark grey **A** nests located in former Leszno Province, medium grey **B** Opole Voivodeship, and light grey **C** Upper Silesia Voivodeship. An arrow indicates the north

Data on ring recoveries and census data were collected between the years 1990 and 2021 mainly in three study areas (Fig. 5): Opolskie Voivodeship (50° 40′ 05.0″ N 17°55′ 22.3″ E), Upper Silesia Voivodeship (50° 16′ 01.8″ N 19° 00′ 55.5″ E) and within the borders of the former Leszno Province in Western Poland (51°5 0′27.9″ N 16° 34′ 32.0″ E). Most of the data were collected by authors; however, some were obtained from the Polish Ringing Scheme database (http://www.stornit.gda.pl/oop_en.php) submitted by volunteers.

**Fig. 6:**
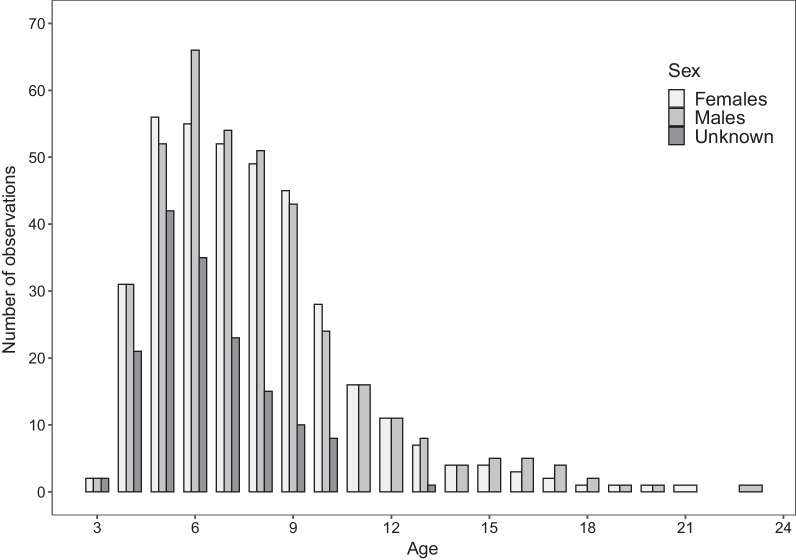
Numbers of observations in each age class, grouped by sex. Age is expressed as a calendar year of life

To limit observation bias, we used only data from study areas where each nest is thoroughly monitored throughout the breeding season. We also excluded data about the birds with certain types of metal rings that were shown to slip from the birds’ legs, and also cases where we knew that the birds were repelled from the nest by people. After excluding incomplete data (see below), we collected 907 records of 372 nests and 330 individuals (Fig. 6). In 23 nests (overall 40 records), we found both partners to be banded. On average, individuals were noted during 2.75 breeding seasons (median = 2, min = 1, max = 9). We collected data on ring recoveries mainly during the beginning of the breeding season (end of March–May). This allowed us to determine the sex of individuals by observing their position during mounting [40]. We visited each nest within the boundaries of three study areas, and if the nest was occupied at any time, the observer checked for rings on *tibiae* and *tarsi*. If the rings were found, the observer tried to read the alphanumerical code until it was successful. Rings were read using spotting scopes or photographs with a telephoto lens. Almost every ring found was eventually read in the case of breeding storks. During the first half of July, we recorded the breeding effect of each pair (including those with rings spotted at the beginning of the breeding season) in the study sites, using a standard census method of counting the number of fledglings standing on the nest, that are considered able to fly [56].

Collected data included nine types of metal and plastic rings with alphanumerical codes. We found no differences in ring recovery probability depending on the ring type (chi-square test of comparisons between all combinations of ring types, p > 0.3).

For each record of a ringed bird occupying a nest in a particular year, we estimated its age (variable Age) using the formula: Year of record − Year of ringing + 1 (except for one individual banded in the second year of life, all birds were ringed as chicks in nests, in two cases birds were banded as adults, therefore, we were unable to calculate their age), geographical coordinates of the nest location and its ID (NestID), breeding performance (Number_of_fledglings—the number of fledglings 1–5, Breeding_success—lack of fledglings 0 or production of any fledglings 1), number of any breeding events on this particular nest (No_repr_events), and nest fidelity in the following year (Fidelity_next_year: 1—returned, 0—not returned). Number of reproductive events was assigned only when the observer was certain about the year of birds first appearance on the nest.

In the case of two records we are certain about the death of an individual, thus these two records and all other records of birds gone missing, that we could not determine as nest switching or death were excluded from the analysis of nest-site fidelity.

We also calculated the average number of fledglings per breeding pair (JZa) in the entire population for each study area for each year of the study. This allowed us to control for the effect of other factors limiting reproductive success that influence the whole local population, like productivity fluctuation [57] or extreme weather events [58]. We calculated the relative productivity (Relative_productivity), i.e. the number of fledglings reduced by the value of JZa of the local population to control for the value of having one fledgling as it differs between years [30].

Data on weather conditions: average monthly minimal temperature and accumulated monthly precipitation in the months March–July, were obtained from TerraClimate ([59], http://www.climatologylab.org/terraclimate.html).

### Spatial analyses

We obtained Corine Land Cover (CLC) datasets from the website of the Chief Inspectorate of Environmental Protection (1990–2018) for data on land cover (see [60] for details), excluding inland waters. The resolution of CLC is too small to identify small rivers, ditches, and ponds. Therefore we decided to use a more exact data source, i.e. The National Database of Topographic Objects (BDOT 10k) in scale 1:10 000, which is part of the National Geodetic and Cartographic Resource. The limitation of these data is that they were collected once, and no historical data are available. However, data on the hydrographic network is particularly important in the case of the white stork, as shallow waters are also used as foraging habitats. We used data on the overall length of small rivers and ditches and the length of the shoreline of reservoirs and wide fragments of rivers (Hydro_length). Both types of land cover (CLC data and BDOT 10k) in 2500 m radius buffers around each nest, using a processing tool in QGIS [61]. For the land cover, we used Corine Land Cover datasets in 1990 for nests existing in 1990–2005, 2006 for nests existing from 2006 to 2011, 2012 for nests existing in 2012–2017 and 2018 for nests existing in 2017–2020. We used the following classes: (1) areas greatly altered by humans (Human_altered), which include continuous urban fabric, discontinuous urban fabric, industrial or commercial units, road and rail networks and associated land, airports**,** mineral extraction sites, dump sites, construction sites, green urban areas and sport and leisure facilities; (2) fruit trees and berry plantations (Permanent_crops); (3) non-irrigated arable land (Arable_land); (4) meadows and pastures (Pastures); (5) other agricultural lands (Other_agri_lands) which include complex cultivation patterns and land principally occupied by agriculture, with significant areas of natural vegetation; (6) forests (Forests) which include broad-leaved forest, coniferous forest, mixed forest and transitional woodland/shrub; (7) sparsely vegetated areas (Sparsely_vegetated_areas); (8) inland marshes (Inland_marshes). For all spatial analyses, we used QGIS 3.16.12 open-source software.

### Statistical analyses

To determine which factors influence the probability of nest-site fidelity, breeding success of white storks, and number of reproductive events on particular nest we used generalized linear mixed models (GLMMs) and linear mixed model (LMM) to determine the factors that affect the number of fledglings. We used binary data for nest-site fidelity (whether the bird was faithful to the nests in the next year or not). Similarly, we used binary data for breeding success (whether the breeding attempt was successful in producing fledglings or not). We used continuous data on the number of fledglings produced (1–5) only for successful nests.

We used bird ID, nest ID and year as random factors in all four models. In the first model (GLMM_1), we included the nest-site fidelity as a dependent variable with a binomial error structure and logit link function. Similarly, in the second model (GLMM_2), we included breeding success as a dependent variable with a binomial error structure and logit link function. In the third model (LMM_3), we included the number of fledglings as a dependent variable with Gaussian error structure and identity link function. In the fourth model, we included number of reproductive events in the nest as a dependent variable with a Poisson error structure (GLMM_4).

In the structure of GLMM_1, we included a quadratic term for age, sex of an individual, breeding success, number of reproductive events on the nest, breeding output relative to the population (number of fledglings for both successful and unsuccessful breeding attempts subtracted by the population mean), the natural logarithm of hydrological network length, the share of human-altered land cover, arable lands, pastures, other agricultural lands within 250 m radius buffer, mean precipitation during the breeding season (PPT_av), and mean minimum temperature during the breeding season (TMIN_av).

In the structure of GLMM_2 and LMM_3, we included a quadratic term for age, presence on the same nest the year before, the interaction between the sex of an individual and the number of reproductive events on the nest, hydrological network length, the share of human-altered land cover, arable lands, pastures, other agricultural lands, mean precipitation during the breeding season (PPT_av), and mean minimum temperature during the breeding season (TMIN_av).

In the structure of GLMM_4 we included a quadratic term for age, sex of an individual, hydrological network length, the share of human-altered land cover, arable lands, pastures and other agricultural.

We also included year, nest ID and bird ID as random factors in the models GLMM_1, GLMM_2, and LMM_3, and nest ID and bird ID as random factors in GLMM_4.

To avoid multicollinearity, we excluded the share of forests from all three models. Multicollinearity in the remaining explanatory variables in both models was not excessive (VIF < 3, [62]). Due to our dataset's extremely small representation of permanent crops, sparsely vegetated areas and inland marshes, we excluded them from all analyses.

We included a quadratic term for age to allow for a nonlinear relationship in all three models, as supported by the improvement of the model AICc score (AICc =  − 4.12; AICc =  − 10.94; AICc =  − 9.31; AICc =  − 116.37, respectively for GLMM_1, GLMM_2, LMM_3, GLMM_4) as calculated with maximum-likelihood estimation.

All analyses were carried out in R 3.3.2 [63]. We used package *DHARMa* [64] to produce diagnostic plots for the final validations of the models. All models were carried out using the *glmmTMB* package [65]. Predictions for the models were made using the *ggeffects* package [66], and the data visualization using the *ggplot2* package [67] and *cowplot* package [68].

### Supplementary Information

**Additional file 1. Table S1.** Results of GLMM for probability of fidelity to the nest in the next year. **Fig. S1.** Effect of age of an individual on the probability of fidelity to nest in the next year.

## Data Availability

The datasets used and analyzed during the current study are available from the corresponding author on reasonable request.
