# Supplementary material for: Age, brood fate, and territory quality affect nest-site fidelity in white stork Ciconia ciconia
Source: Front Zool. 2023 Sep 21;20:33. doi: 10.1186/s12983-023-00506-y (PMC10512545; doi:10.1186/s12983-023-00506-y)

**Age, brood fate, and territory quality affect nest-site fidelity in White Stork *Ciconia ciconia***

Joanna T. Bialas^1^*, Joachim Siekiera^2^, Artur Siekiera^2^, Wiesław Chromik^3^, Łukasz Dylewski^1^, Marcin Tobolka^1,4^

^1^ Department of Zoology, Poznań University of Life Sciences, Wojska Polskiego 71C, 60-625 Poznań, Poland

^2^Żywocice, Poland

^3^Katowice, Poland

^4^ Konrad Lorenz Institute of Ethology, University of Veterinary Medicine Vienna, Savoyenstraβe 1a, 1160 Wien, Austria

*Correspondence: jtwozna@gmail.com

**Supplementary materials**

**Table S1 Results of GLMM for probability of fidelity to the nest in the next year.**

|  | Estimate | SE | p |  |
| --- | --- | --- | --- | --- |
| (Intercept) | -0.91 | 3.45 | 0.79 | |
| Age | 0.45 | 0.20 | **0.03** | |
| Age^2 | -0.02 | 0.01 | **0.05** | |
| Breeding_success | 1.58 | 0.42 | **0.00** | |
| No_repr_events | 0.26 | 0.13 | **0.04** | |
| Relative_productivity | 0.11 | 0.15 | 0.45 | |
| Hydro_network | -0.29 | 0.26 | 0.26 | |
| Human_altered | 2.90 | 1.81 | 0.11 | |
| Arable_land | 1.16 | 0.83 | 0.16 | |
| Pastures | 3.80 | 1.73 | **0.03** | |
| Other_agri_lands | 4.10 | 2.08 | **0.05** | |
| PPT_av | 0.01 | 0.01 | 0.38 | |
| TMIN_av | 0.00 | 0.17 | 0.98 | |

The explanatory variables were age and its quadratic term (Age, Age^2 respectively), breeding success (Breeding_success), number of reproductive events of an individual on the particular nest (No_repr_events), productivity relative to the population mean (Relative_productivity), natural logarithm of length of hydrological network (Hydro_network), cover of human-altered habitat (Human_altered), cover of arable lands (Arable_land), cover of pastures and meadows (Pastures), cover of other agricultural lands (Other_agri_lands), average precipitation (PPT_av) and minimum temperature (TMIN_av) during the breeding season. For binary variables breeding success and sex the presented values are for consecutively presence of success and males, as lack of success and females are treated as a reference (the estimates B are equal to 0). Significant results (p<0.05) are in bold.

**Fig. S1 Effect of age of an individual on the probability of fidelity to nest in the next year.** Predictions resulted from generalized mixed model with binomial distribution. Transparent dots are observed values, line indicates predictions from the model and grey areas represent 95% CI. Predictions are calculated for the whole range of age classes.


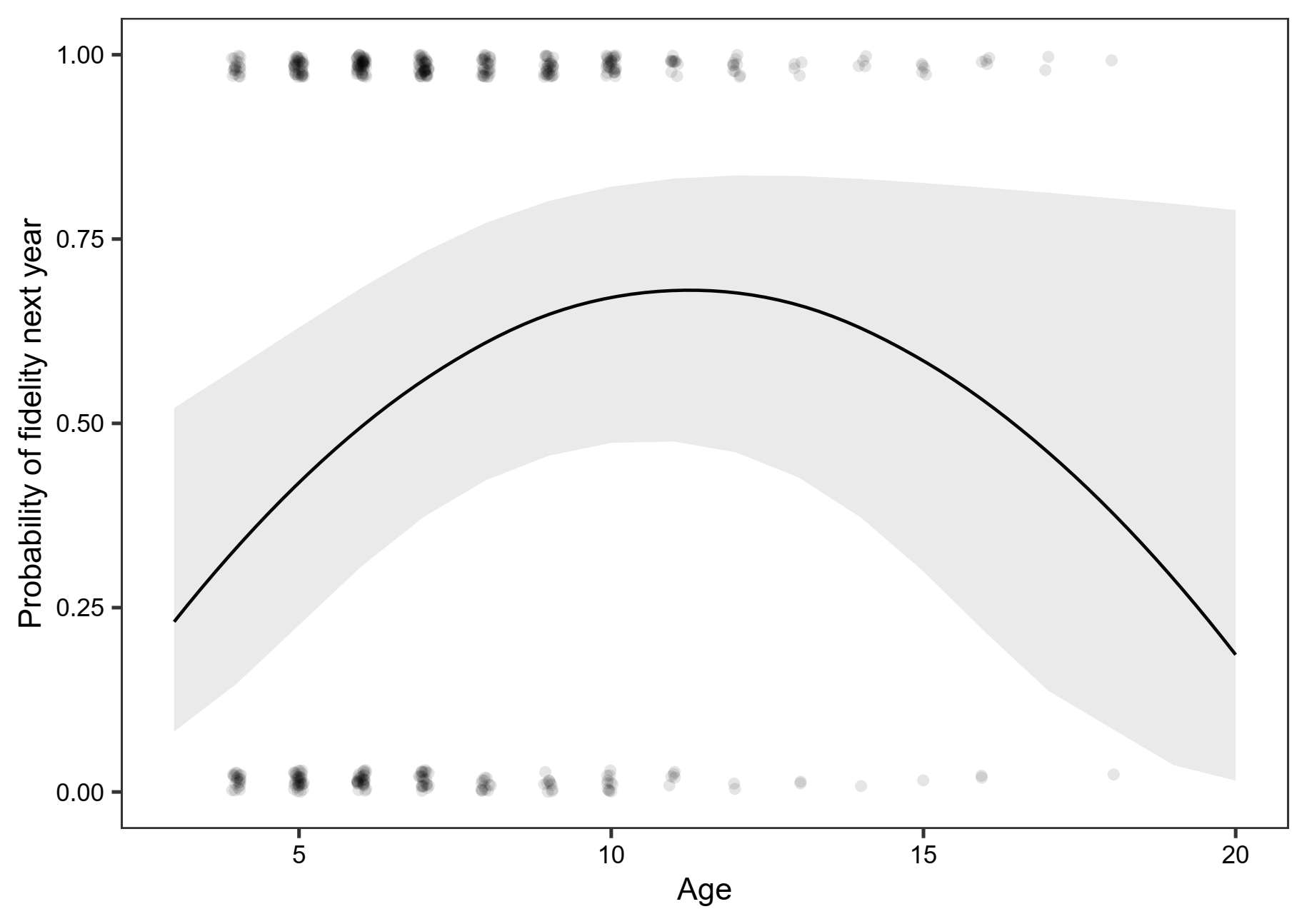

Supplement: Supplementary file 1 — Additional file 1. Table S1. Results of GLMM for probability of fidelity to the nest in the next year. Fig. S1. Effect of age of an individual on the probability of fidelity to nest in the next year. [file 12983_2023_506_MOESM1_ESM.docx]
